# Increased CCR7^lo^PD-1^hi^CXCR5^+^CD4^+^ T Cells in Peripheral Blood Mononuclear Cells Are Correlated with Immune Activation in Patients with Chronic HBV Infection

**DOI:** 10.1155/2018/1020925

**Published:** 2018-10-08

**Authors:** Ya-Xin Huang, Qi-Yi Zhao, Li-Li Wu, Dong-Ying Xie, Zhi-Liang Gao, Hong Deng

**Affiliations:** ^1^Department of Infectious Diseases, The Third Affiliated Hospital of Sun Yat-sen University, Guangzhou, Guangdong 510630, China; ^2^Guangdong Key Laboratory of Liver Disease Research, The Third Affiliated Hospital of Sun Yat-sen University, Guangzhou 510630, China; ^3^Key Laboratory of Tropical Disease Control (Sun Yat-sen University), Ministry of Education, Guangzhou, Guangdong 510080, China

## Abstract

T follicular helper cells (Tfh cells) affect essential immune pathogenesis in chronic hepatitis B virus (HBV) infection. The CCR7^lo^PD-1^hi^ Tfh subset has a partial Tfh effector phenotype and is associated with active Tfh differentiation, whereas the CCR7^hi^PD-1^lo^ Tfh subset is a resting phenotype. We recruited 20 healthy volunteers and 77 patients with chronic HBV infection, including those in the immune tolerant (IT) phase (n=19), immune clearance (IC) phase (n=20), low replicative (LR) phase (n=18), and reactivation (RA) phase (n=20). The expression of CD4, CXCR5, PD-1, and CCR7 was detected in T cells from peripheral blood by flow cytometry. The frequency of the CCR7^lo^PD-1^hi^ T subset was significantly higher in the patients than in the healthy controls (14.92±4.87% vs 12.23±2.95%, p=0.018). The frequency of this Tfh subset in the IC group (18.42%±3.08) was increased compared with the IT group (11.94±2.87%, p=0.001) and LR group (13.65±4.93%, p=0.031) and was higher in the RA group than in the IT group (16.03±5.37% vs 11.94±2.87%, p=0.030). We observed a weak positive correlation between the CCR7^lo^PD-1^hi^ Tfh subset population and the alanine transaminase (ALT) level (r=0.370, p=0.001). The CCR7^lo^PD-1^h^ Tfh subset in the chronic HBV-infected patients was elevated to various degrees among the different immune phases. CCR7^lo^PD-1^hi^CXCR5^+^CD4^+^ T cells are correlated with the immune status of chronic HBV infection patients and may be developed as a potential indicator for antiviral treatment.

## 1. Introduction

HBV infection remains among the most serious issues in global public health despite extensive vaccination and effective antiviral treatments. A total of 250 million people suffer from chronic hepatitis B virus (HBV) infection worldwide, most of whom live in Africa and Asia [1, 2]. HBV-associated diseases, such as liver failure, cirrhosis, and hepatocellular carcinoma, contribute to the deaths of 1 million people per year [3].

Our understanding of the natural history of HBV infection and the resultant disease is continuously improving. Complex interactions between the viral and host immune systems participate in disease progression, allowing for HBV penetration into host cells, formation of persistence, and chronization of HBV infection or complete elimination of the virus [4, 5]. Although various clinical and experimental investigations have helped diagnose, treat, and prevent hepatitis B, the exact mechanism underlying the host immune reactions remains unclear.

According to the complex interactions between the virus, hepatocytes, and the host immune system, the natural course of chronic HBV infection is usually stratified into 4 phases, the immune tolerant (IT) phase, the immune clearance (IC) phase, the low replicative (LR) phase, and the reactivation (RA) phase [6].

Proteins of partial HBV can modulate immunity and enable immune escape. In the course of the disease, a better prognosis can be achieved if HBeAg seroconversion occurs early. The prevalence of cirrhosis and hepatocellular carcinoma in patients during this time declines. In addition, HBsAg loss and/or seroconversion is considered the ideal goal of treatment and a milestone in effective treatment response in both HBeAg-positive and HBeAg-negative patients [7].

The production of antibodies plays an indispensable role in both HBeAg and HBsAg seroconversion[8]. Circulating CXCR5^+^CD4^+^ T cells, which are the counterpart of T follicular helper (Tfh) cells in the peripheral blood, have been reported to play a significant role in accelerating HBeAg seroconversion in chronic HBV-infected patients [9].

Tfh cells are considered to be a subset of CD4^+^ T cells in secondary lymphoid tissues that express CXC-chemokine receptor 5 (CXCR5), which helps Tfh cells localize to B cell follicles. Studies have reported that CXCR5^+^CD4^+^T cells are more efficient than CXCR5^−^CD4^+^ T cells in inducing B cells to secrete antibodies and switch antibody classes [10–12]. Tfh cells coexpress programmed cell death protein 1 (PD-1) and inducible T cell co-stimulator (ICOS) and downregulate CC-chemokine receptor 7 (CCR7) [13–15]. Several investigations have found elevated expression of circulating CXCR5^+^CD4^+^ T cells in patients with autoimmune diseases (such as systemic lupus erythematosus (SLE) and Sjogren's syndrome)[16, 17] and infectious diseases (such as hepatitis B and C)[18, 19]. However, He J et al. found no increase in the frequency of circulating CXCR5^+^CD4^+^ T cells in SLE patients [20], which was inconsistent with previous investigations. In addition, a study showed that there was no difference in the circulating CXCR5^+^CD4^+^ T cell frequency between healthy controls and HCV patients. Interestingly, this study also found that CXCR5^+^CD4^+^ T cells were efficient in supporting B cell responses [21]. Based on current evidence, there is no clear correlation between the activity of CXCR5^+^CD4^+^ T cells and their frequency in peripheral blood.

Tfh cells are comprised of various subsets with different phenotypes and functions [22]. He J et al. reported that CCR7^lo^PD-1^hi^CXCR5^+^CD4^+^ T cells have a partial Tfh effector phenotype exhibiting active Tfh differentiation in lymphoid tissues. In contrast, the CCR7^hi^PD-1^lo^ Tfh subset has a resting phenotype [20]. Studies in mice found that CXCR5^hi^PD-1^hi^ germinal center Tfh cells likely downregulate CXCR5, PD-1, and BCL-6, re-express CCR7, IL-7R*α*, and CD62L, and thus differentiate into memory cells and persist for a long time [22, 23]. IL-7 possibly increases the level of Tfh cells in the patients with chronic hepatitis B[24]. Studies investigating cystic echinococcosis also reported that CCR7^lo^PD-1^hi^CXCR5^+^CD4^+^ T cells were increased in patients [25].

The CCR7^lo^PD-1^hi^ and CCR7^hi^PD-1^lo^ Tfh subsets in the peripheral blood have not been comprehensively investigated during the complex immunologic progression of chronic HBV infection. We hypothesize that these two Tfh subsets play a larger role in the immune response of chronic HBV infection than Tfh cells, containing multifarious subsets. The objective of this study was to detect the frequencies of CCR7^lo^PD-1^hi^CXCR5^+^CD4^+^ T cells and CCR7^hi^PD-1^lo^CXCR5^+^CD4^+^ T cells in peripheral blood mononuclear cells (PBMCs) from patients with chronic HBV infection and compare these frequencies to those in non-HBV infected controls. Furthermore, the correlations between the frequencies of the two subsets and alanine transaminase (ALT), which is the consequence of HBV replication, and the HBsAg level were evaluated. These findings provide new insights into the correlation between the frequencies of the two CXCR5^+^CD4^+^ T subsets and the immune reaction in chronic HBV infection.

## 2. Materials and Methods

### 2.1. Patients and Controls

A total of 77 patients with chronic HBV infection were recruited from the Third Affiliated Hospital of Sun Yat-sen University (Guangzhou, China) for this cross-sectional study. These patients were HBsAg-seropositive for longer than 6 months. The patients were divided into immune tolerant (IT) phase group (n=19), immune clearance (IC) phase group (n=20), low replicative (LR) phase group (n=18), and reactivation (RA) phase group (n=20) according to the Asian Pacific Association for the Study of Liver guidelines [6]. In addition, 20 healthy individuals were enrolled from the physical examination center. All healthy individuals were non-HBV infected, HCV infected, or HIV infected and tested normal for ALT and aspartate aminotransferase (AST).

The exclusion criteria for this study included coinfection with hepatitis viruses A, C, D, or E or HIV. Patients with autoimmune diseases, drug-induced liver injury, decompensated or compensated cirrhosis, malignant comorbidities within the prior 5 years, or previous antiviral or immunomodulatory drug treatments were also excluded.

This study was approved by the Human Ethics Committee of the Third Affiliated Hospital of Sun Yat-sen University (Guangzhou, China) and conducted in accordance with the Declaration of Helsinki guidelines. All subjects provided written informed consent before collecting the blood samples.

### 2.2. Peripheral Blood Mononuclear Cell Separation

Peripheral venous blood samples were collected from all subjects into 5 mL tubes containing EDTA as the anticoagulant. Within 4 hours of the collection, the PBMCs were separated from the samples by Ficoll separation (Axis-Shield PoC AS, Oslo, Norway). Approximately 5 × 10^∧^6 PBMCs were collected from each sample and frozen at -80°C until analysis.

### 2.3. Analysis of Cell Surface Molecule Expression by Flow Cytometry

The cells were thawed and incubated at 37°C and 5% CO_2_ in RPMI-1640 with 10% FCS (cell culture media) for 4 hours. Then, the cells were stained with anti-CD3 FITC (clone:SK7, eBioscience, San Diego, CA, USA), anti-CD4 eFluor® (clone: OKT4, eBioscience, San Diego, CA, USA), anti-CXCR5 APC (clone: MU5UBEE, eBioscience, San Diego, CA, USA), anti-PD-1 PE-Cy7 (clone: J105, eBioscience, San Diego, CA, USA), anti-CCR7 PE (clone: 3D12, eBioscience, San Diego, CA, USA), and isotype antibodies (eBioscience, San Diego, CA, USA). The cells were washed, and the marker expression was detected by flow cytometry (Beckman Gallios Coulter, Inc., CA, USA). The samples underwent detection within 4 hours. The data were analyzed using FlowJo 10.0 (Tree Star Inc., Ashland, Or, USA).

### 2.4. Laboratory Indices

The quantitative values of the following indices were tested by Elecsys (Roche Diagnostics GmbH, Mannheim, Germany) at the noted reference ranges: HBsAb, 0 - 10 IU/L; HBeAg, <1.0 cut-off index (COI); HBeAb, >1.0 COI; and HBcAb, >1.0 COI. The HBsAg titers were quantified using Elecsys HBsAg II Quant reagent kits (Roche Diagnostics, Indianapolis, IN, USA). The detection limit of the kit was 20 IU/mL. The HBV-DNA levels were quantitated by performing real-time quantitative polymerase chain reaction (Daan GENE, Guangzhou, China). The detection limit of the assay was 100 IU/mL. The biochemical indices were detected using an autobiochemical analyzer (HITACHI 7180, Tokyo, Japan). ALT and AST were within the reference ranges of 3-35 U/L and 13-35 U/L, respectively.

### 2.5. Statistical Analysis

All statistical analyses were performed using SPSS 24.0 software for Windows (SPSS Inc., Chicago, IL, USA), and the data were presented as the median (minimum, maximum) (age, ALT, AST, HBV DNA, and HBsAg) or the mean ± standard deviation (frequencies of cells). Multiple comparisons were performed using nonparametric Kruskal-Wallis tests with Bonferroni correction for the sub-analyses. The statistical significance between two groups was determined by performing a Mann–Whitney U test. The correlation between the CCR7^lo^PD-1^hi^CXCR5^+^CD4^+^ T cell frequency and clinical parameters was examined by performing Spearman's rank correlation. All statistical tests were two-tailed. The differences were considered statistically significant at p<0.050.

## 3. Results

### 3.1. Study Subjects' Characteristics

The subjects in this study included 77 treatment-naive patients who had been HBsAg-positive for longer than 6 months and 20 healthy volunteers with normal ALT and AST (Table 1). According to the immune phases, the 77 HBV-infected patients were further classified as follows: IT phase (n=19), IC phase (n=20), LR phase (n=18), and RA phase (n=20) (Table 2).

### 3.2. Frequencies of Circulating CXCR5^+^CD4^+^ T Cells and Subsets in Peripheral Blood Mononuclear Cells

**Figure 1:**
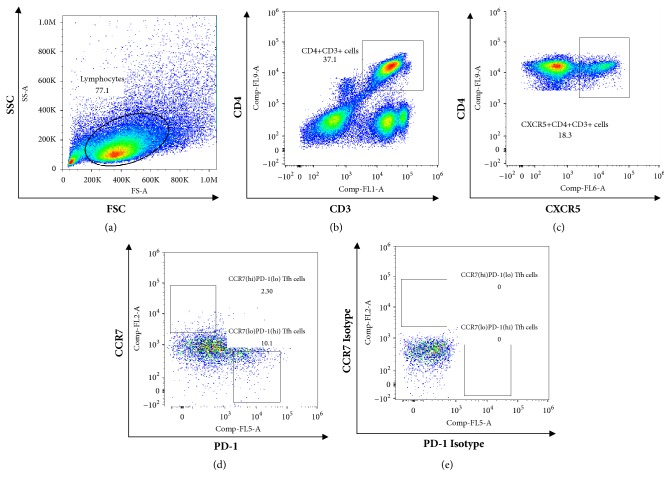
Gating strategy and representative dot plots of CCR7 and PD-1 expression on CXCR5^+^CD4^+^ T cells from chronic HBV infected patients. (a) Living lymphocytes gate. (b) CD4^+^CD3^+^ cells gate. (c) CXCR5^+^CD4^+^CD3^+^ cells gate. (d) CCR7^lo^PD-1^hi^ Tfh cells gate and CCR7^hi^PD-1^lo^ Tfh cells gate were set according the isotypes (e). At least approximately 100,000 events were analyzed in each sample.

**Figure 2:**
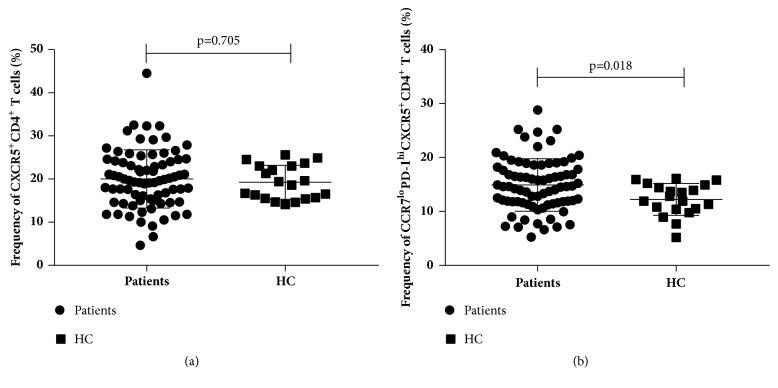
Frequencies of CXCR5^+^CD4^+^ T cells and CCR7^lo^PD-1^hi^ CXCR5^+^CD4^+^ T cells in chronic HBV patients (n=77) and healthy controls (n=20). (a) Frequency of CXCR5^+^CD4^+^ among all CD4^+^CD3^+^ cells. (b) Frequency of CCR7^lo^PD-1^hi^CXCR5^+^CD4^+^ among all CXCR5^+^CD4^+^CD3^+^ cells. Horizontal lines show the median.

The frequency of circulating CXCR5^+^CD4^+^ T cells in the PBMC samples was detected by flow cytometry (Figure 1). The CXCR5^+^CD4^+^ T cell frequency in the patients with chronic HBV infection was higher than that in the non-HBV infected individuals, but not significantly (20.01±6.76% vs 19.26±3.93%, p=0.705, Figure 2(a)). Nevertheless, the frequency of CCR7^lo^PD-1^hi^ CXCR5^+^CD4^+^ T cells was significantly higher in the patients than in the healthy controls (14.92±4.87% vs 12.23±2.95%, p=0.018, Figure 2(b)). In addition, the frequency of the CCR7^hi^PD-1^lo^ CXCR5^+^CD4^+^ T cells was lower in the patients with chronic HBV infection, but not significantly (p=0.715).

**Figure 3:**
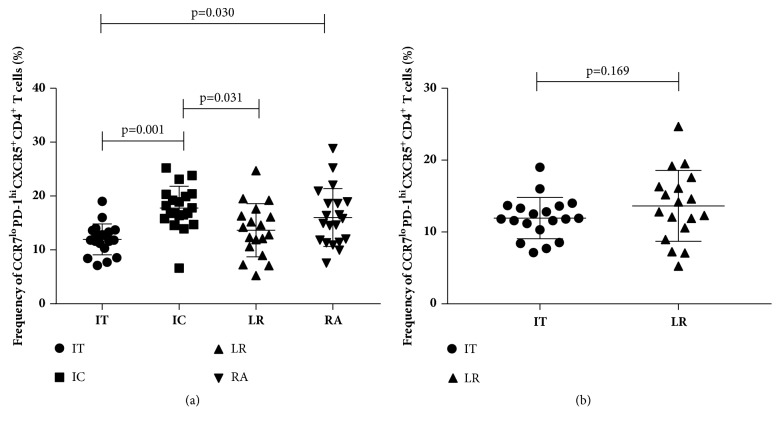
Frequency of CCR7^lo^PD-1^hi^CXCR5^+^CD4^+^ T cells among all CXCR5^+^CD4^+^CD3^+^ cells in the patients with chronic HBV infection and between IT group and LR group. (a)Differences between the IT group (n=19) and IC group (n=20), between the IT group and RA group (n=20), and between the IC group and LR group (n=18) were significant. Statistical comparison was performed using a Bonferroni correction. (b)Difference between the IT group (n=19) and LR group (n=18) was not significant (p=0.169). The horizontal lines show the median.

We further investigated the association among the frequencies of the CXCR5^+^CD4^+^ T cells, CCR7^lo^PD-1^hi^ Tfh subset, and HBV by stratifying the patients according to their immune status (IT, IC, LR, or RA). Based on the Kruskal-Wallis tests, although no significant difference was observed in the frequency of the CXCR5^+^CD4^+^ T cells among the 4 groups (p=0.885), differences in the CCR7^lo^PD-1^hi^ Tfh subset were observed in the groups (p<0.001). After conducting the Bonferroni correction, we found that the frequency of CCR7^lo^PD-1^hi^ CXCR5^+^CD4^+^ T cells was higher in the IC group (18.42%±3.08) than in the IT group (11.94±2.87%, p=0.001) and LR group (13.65±4.93%, p=0.031). In addition, the frequency of the CCR7^lo^PD-1^hi^ CXCR5^+^CD4^+^ T cells was higher in the RA group than in the IT group (16.03±5.37% vs 11.94±2.87%, p=0.030, Figure 3(a)). Although frequency of CCR7^lo^PD-1^hi^ CXCR5^+^CD4^+^ T cells of IT group was lower than LR group, the difference was not significant (11.941±2.868 % vs 13.648±4.930%, p=0.169) (Figure 3(b)).

**Figure 4:**
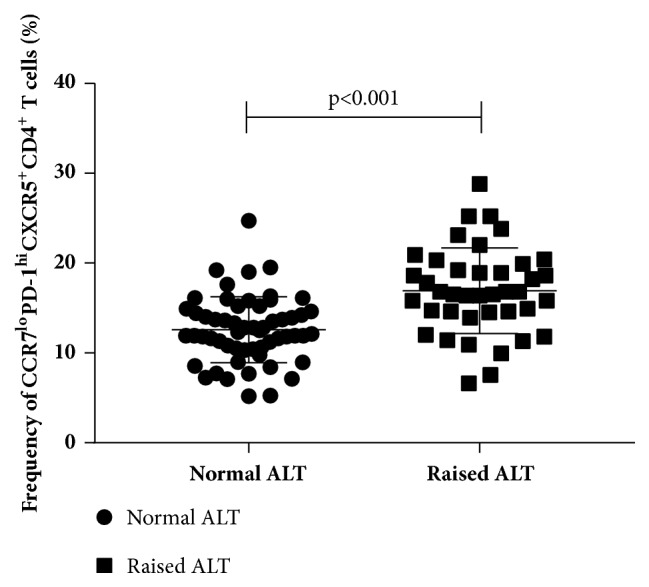
Frequencies of CCR7^lo^PD-1^hi^CXCR5^+^CD4^+^ T cells among all CXCR5^+^CD4^+^CD3^+^ cells in the people with normal ALT and raised ALT. Difference between two group was significant (p<0.001). The horizontal lines show the median.

The comparison between people with raised ALT and normal ALT has been conducted and the difference was significant (16.91±4.77% vs 12.58±3.68%, p<0.001). The frequency of the CCR7^lo^PD-1^hi^ CXCR5^+^CD4^+^ T cells was higher in the raised ALT group relative to the normal ALT group (Figure 4).

### 3.3. Correlation between the Two Tfh Cell Subsets and Clinical Parameters of the Chronic HBV Infected Patients

**Figure 5:**
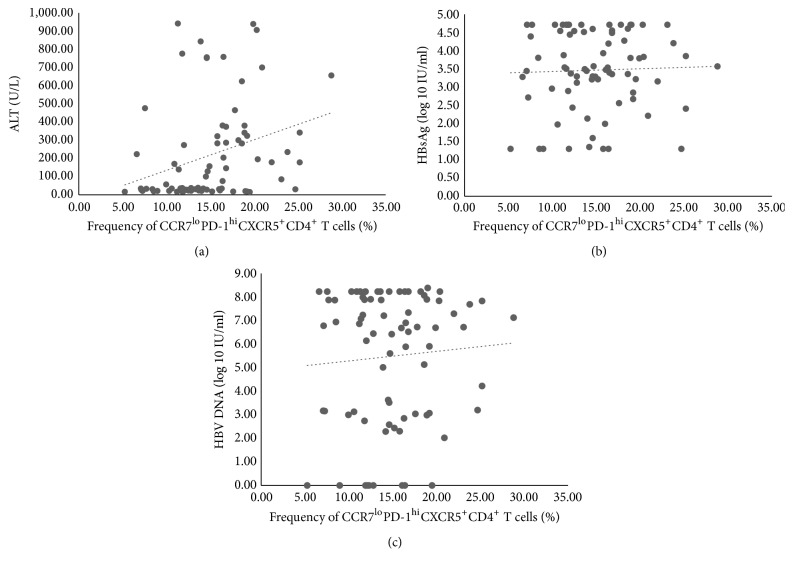
Correlations between frequency of CCR7^lo^PD-1^hi^CXCR5^+^CD4^+^ T cells and clinical indices. (a) Correlation between frequency of CCR7^lo^PD-1^hi^ Tfh cells and ALT level (r=0.370, p=0.001). (b) Correlation between frequency of CCR7^lo^PD-1^hi^ Tfh cells and HBV DNA load (p>0.05). (c) Correlation between frequency of CCR7^lo^PD-1^hi^ Tfh cells and HBsAg (p>0.05). Statistical comparison was performed using Spearman's rank correlation analysis.

The correlations between the CCR7^lo^PD-1^hi^CXCR5^+^CD4^+^ T cell and the CCR7^hi^PD-1^lo^CXCR5^+^CD4^+^ T cell populations in the PBMCs and the patients' ALT, HBV DNA load, HBsAg level, age, and gender were investigated. Based on Spearman's rank correlation analysis, there was a positive correlation between the CCR7^lo^PD-1^hi^CXCR5^+^CD4^+^ T cell populations and levels of ALT (r=0.370, p=0.001, Figure 5(a)). However, the correlation was weak and not convincing enough. Besides, no correlation was observed between the frequency of the CCR7^hi^PD-1^lo^CXCR5^+^CD4^+^ T cells and the ALT levels (r=-0.143, p>0.050). Furthermore, CCR7^lo^PD-1^hi^CXCR5^+^CD4^+^ T cells (r=-0.028, p>0.005, Figure 5(b)) or CCR7^hi^PD-1^lo^CXCR5^+^CD4^+^ T cells (r=-0.160, p>0.005) had no correlation with HBV DNA. Neither of the CCR7^lo^PD-1^hi^ (r= 0.008, p>0.050, Figure 5(c)) or the CCR7^hi^PD-1^lo^ Tfh subsets (p>0.050) were correlated with HBsAg.

Although a negative correlation was observed between the CCR7^lo^PD-1^hi^CXCR5^+^CD4^+^ T cells and age in the patients with chronic HBV infection (r=-0.264, p=0.020), no evidence was found supporting a correlation between the CCR7^hi^PD-1^lo^CXCR5^+^CD4^+^ T cells and age among the patients (r=0.182, p=0.114). Further analysis showed significant difference in ages among the patients (p=0.002), and younger subjects were more likely to be in the IT and IC than in the LR and RA phases. However, no significant difference was detected in age and frequency of CCR7^hi^PD-1^lo^CXCR5^+^CD4^+^ T cells among the healthy controls. Furthermore, no difference was observed in the gender ratios in either the patients or healthy controls.

Correlations among the two Tfh subsets and clinical characteristics were analyzed in each subgroup of patients with chronic HBV infection. However, no significant result was observed (Tables 3 and 4).

## 4. Discussion

The host immune mechanism, including innate and adaptive immunity, is important in the pathogenesis of hepatitis B. HBeAg seroconversion, led by viral and host immunity, is essential for the progression of chronic HBV infection, which is associated with a reduced risk of progressive liver inflammation, liver cirrhosis and liver cancer [6, 26]. In the natural history of chronic HBV infection, patients who have successfully undergone seroconversion usually become inactive HBsAg carriers with positive anti-HBeAg in the blood [27, 28]. A high frequency of circulating CXCR5^+^CD4^+^ T cells has been shown to promote HBeAg seroconversion in chronic HBV patients [7].

The main function of Tfh cells is to support B cell maturation and differentiation. Tfh cells and B cells repeatedly intimately interact in the germinal center, where Tfh cells deliver important survival and differentiation signals to B cells that participate in the affinity, antibody isotype class, and potency of the ensuing antibody response [29]. Nevertheless, various studies have reported conflicting results. Many research studies have found increased expression of circulating Tfh cells in SLE, but no increase in the frequency of circulating CXCR5^+^CD4^+^ T cells has also been reported in a study investigating SLE [20].

Our study found that the circulating CXCR5^+^CD4^+^ T cell frequency was higher, but not significantly, in patients with chronic HBV infection than in non-HBV infected individuals. This finding is inconsistent with several former studies. The frequency of the CXCR5^+^CD4^+^ T cells was not accurate enough to describe the difference in the immune response between chronic HBV infected patients and healthy people.

Heterogeneity is observed in Tfh cells [30]. According to the CCR7 and PD-1 expression on the cell surface, two major subsets were clearly identified within the circulating CXCR5^+^CD4^+^ T cells. A study in repeated implantation failure reported the proportion of CCR7^lo^PD-1^hi^CXCR5^+^CD4^+^ T cells was positively correlated with IL-21[31]. Another study found IL-21 was positively correlated with CCR7^lo^PD-1^hi^ Tfh subset in transitional phase of Cystic echinococcosis[25].IL-21 is the main cytokine secreted by Tfh cells, reported as a critical immunomodulatory cytokine with various effects on all populations of lymphocytes. It can promote Tfh cells differentiation, regulate B cells differentiation and proliferation, induce plasma cell differentiation and immunoglobulin production[32].Our investigation demonstrated that the frequency of the CCR7^lo^PD-1^hi^ subset was increased in the chronic HBV infected patients and positively correlated with ALT on a weak level. Most peripheral CXCR5^+^CD4^+^ T cells are resting cells, and a very small population expressing ICOS and very high levels of PD-1 are activated cells [33]. He J et al showed that the CCR7^lo^PD-1^hi^ subset had a Tfh precursor phenotype, whereas the phenotype of the CCR7^hi^PD-1^lo^ subset was characteristic of resting cells [20].

PD-1 is a negative regulatory molecule that becomes up-regulated on activated T cells, B cells, monocytes, natural killer cells and dendritic cells and is particularly highly expressed on Tfh cells. The PD-1 ligands, i.e., PD-L1 and PD-L2, are extensively expressed on various cells, including T cells, B cells, dendritic cells and macrophages [34, 35]. CCR7 is a homing molecule expressed on the T cell surface and is essential for the migration of naive T cells through specialized high endothelial venules (HEVs). In addition, B cells exploit CCR7 to efficiently enter lymphoid nodes[36–38].

In our study, a stronger immune response was observed in the chronic HBV infected patients with a higher frequency of the CCR7^lo^PD-1^hi^ Tfh subset compared to the healthy controls. We hypothesize that the CCR7^lo^PD-1^hi^ Tfh subset, as an effector phenotype, may contribute to the immune response in chronic HBV infection. Furthermore, in chronic HBV infection, the frequency of the CCR7^lo^PD-1^hi^ Tfh subset is higher in the IC phase than in the IT and LR phases, suggesting that CCR7^lo^PD-1^hi^CXCR5^+^CD4^+^ T cells may indicate the level of the immune response more precisely than general CXCR5^+^CD4^+^ T cells and are related to immune status. This hypothesis was further supported by the higher frequency of the CCR7^lo^PD-1^hi^ Tfh subset in the RA phase than in the IT phase. However, there was no significant difference of frequency of CCR7^lo^PD-1^hi^ Tfh subset between IT and LR group was observed. More investigation should be complemented.

Currently, the indications for antiviral treatment in chronic HBV infection are mainly based on a combination of the following three criteria: HBV DNA load, ALT levels, and severity of liver disease [6]. By preventing the progression of liver disease and early liver-related deaths, timely and valid therapy could be highly beneficial for improving quality of life and survival [39]. We speculate that the frequency of the CCR7^lo^PD-1^hi^ Tfh subset in the blood, which was correlated to the immune status in chronic HBV infection, may help physicians determine when to initiate antiviral treatment. Certainly, further investigations are needed to study factors influencing the frequency of CCR7^lo^PD-1^hi^CXCR5^+^CD4^+^ T cells.

## 5. Conclusion

In conclusion, the CCR7^lo^PD-1^hi^CXCR5^+^CD4^+^ T cell frequency is higher in patients with chronic HBV infection than in healthy individuals and is positively correlated with serum ALT levels in patients, indicating that CCR7^lo^PD-1^hi^CXCR5^+^CD4^+^ T cells may be involved in HBV-related immune responses. Moreover, different frequencies of CCR7^lo^PD-1^hi^CXCR5^+^CD4^+^ T cells are observed in patients at different immune phases. These findings might improve our understanding of the immunological pathogenesis of chronic HBV infection and may provide a novel indication for antiviral treatment.

## Figures and Tables

**Table 1:** Clinical characteristics of the patients with chronic HBV infection and healthy controls.

	Patients	HC	p-value
Subjects, n	77	20	/
Gender, males/females	45/32	10/10	0.499
Age, y	35(18-71)	27(18-52)	0.053
ALT, IU/L	75(15-942)	15(7-31)	<0.001
AST, IU/L	51(13-915)	14.5(7-34)	<0.001
HBV DNA, log10 IU/mL	6.72(0-8.38)	NA	/
HBsAg, log10 IU/mL	3.50(1.30-4.72)	NA	/
HBeAg, positive/negative, n	39/38	0/20	<0.001
ALT, elevated/normal, n	40/37	0/20	<0.001

*Abbreviations.* ALT: alanine transaminase; AST: aspartate aminotransferase; HBeAg: hepatitis B e antigen; HBsAg: hepatitis B s antigen; HBV: hepatitis B virus; HC: healthy controls; NA: not applicable.

(a) Values are expressed as the median (minimum-maximum) for age, ALT, AST, and HBV DNA.

(b) HBV DNA<100 was treated as 0 log10 IU/ml.

**Table 2:** Clinical characteristics of 4 subgroups of patients with chronic HBV infection.

	IT	IC	LR	RA	p-value
Subjects, n	19	20	18	20	/
Gender, males/females	10/9	12/8	11/7	12/8	0.950
Age, y	29(18-52)	29(18-56)	41.5(18-71)	42(24-65)	0.002
ALT, IU/L	27(16-38)	293(85-939)	25.5(15-34)	332(57-942)	<0.001
AST, IU/L	28(13-38)	129(51-491)	22(19-34)	177.5(39-915)	<0.001
HBV DNA, log10 IU/mL	7.87(6.45-8.38)	7.76(3.72-8.23)	2.36(0-3.20)	5.51(2.00-8.23)	<0.001
HBsAg, log10 IU/mL	4.557(3.495-4.54)	3.85(2.85-4.60)	2.69(1.35-3.53)	3.32(1.30-4.61)	<0.001
HBeAg, positive/negative, n	19/0	20/0	0/18	0/20	<0.001
ALT, elevated/normal, n	0/19	20/0	0/18	20/0	<0.001

(a) Values are expressed as median (minimum-maximum) for age, ALT, AST, and HBV DNA.

(b) HBV DNA<100 was treated as 0 log10 IU/ml.

(c) HBsAg<20 was treated as 20.

**Table 3:** Correlations between frequency of CCR7^lo^PD-1^hi^CXCR5^+^CD4^+^ T cells and clinical characteristics in 4 subgroups of patients with chronic HBV infection.

Group/Clinical characteristics	ALT(U/L)		HBV DNA, log10 IU/ml		HBsAg, log10IU/ml	
	r-value	p-value	r-value	p-value	r-value	p-value
IT	0.022	0.930	0.238	0.326	-0.300	0.213
IC	-0.149	0.530	-0.145	0.543	0.192	0.461
LR	-0.206	0.412	0.145	0.567	0.220	0.381
RA	0.189	0.425	-0.222	0.348	-0.273	0.258

**Table 4:** Correlations between frequency of CCR7^hi^PD-1^lo^CXCR5^+^CD4^+^ T cells and clinical characteristics in 4 subgroups of patients with chronic HBV infection.

Group/Clinical characteristics	ALT(U/L)		HBV DNA, log10 IU/ml		HBsAg, log10IU/ml	
	r-value	p-value	r-value	p-value	r-value	p-value
IT	-0.069	0.780	-0.344	0.149	-0.067	0.784
IC	0.171	0.470	-0.384	0.095	0.235	0.363
LR	0.065	0.797	-0.072	0.777	-0.156	0.536
RA	0.287	0.220	0.087	0.716	0.055	0.822

## Data Availability

The data used to support the findings of this study are available from the corresponding author upon request.
